# Metabolism-associated genome-wide epigenetic changes in bovine oocytes during early lactation

**DOI:** 10.1038/s41598-020-59410-8

**Published:** 2020-02-11

**Authors:** Mikhael Poirier, Dawit Tesfaye, Tsige Hailay, Dessie Salilew-Wondim, Samuel Gebremedhn, Franca Rings, Christiane Neuhoff, Karl Schellander, Michael Hoelker

**Affiliations:** 1 Institute of Animal Science, Animal Breeding and Husbandry Group, University of Bonn, Bonn, Germany; 2 Department of Biomedical Sciences, Animal Reproduction and Biotechnology Laboratory, Colorado State University, Fort Collins, CO United States of America

**Keywords:** Epigenetic memory, Risk factors

## Abstract

Dietary intake in early lactating cows is outmatched by milk production. These cows experience a negative energy balance, resulting in a distinct blood metabolism and poor reproductive function due to impaired ovulation and increased embryo loss. We hypothesize that oocytes from lactating cows undergoing transient metabolic stress exhibit a different epigenetic profile crucial for developmental competence. To investigate this, we collected oocytes from metabolically-profiled cows at early- and mid-postpartum stages and characterized their epigenetic landscape compared with control heifers using whole-genome bisulfite sequencing. Early-postpartum cows were metabolically deficient with a significantly lower energy balance and significantly higher concentrations of non-esterified fatty acids and beta-hydroxybutyrate than mid-postpartum animals and control heifers. Accordingly, 32,990 early-postpartum-specific differentially methylated regions (DMRs) were found in genes involved in metabolic pathways, carbon metabolism, and fatty acid metabolism, likely descriptive of the epigenetic regulation of metabolism in early-postpartum oocytes. DMRs found overlapping CpG islands and exons of imprinted genes such as *MEST* and *GNAS* in early-postpartum oocytes suggest that early lactation metabolic stress may affect imprint acquisition, which could explain the embryo loss. This whole-genome approach introduces potential candidate genes governing the link between metabolic stress and the reproductive outcome of oocytes.

## Introduction

In previous decades, genetic selection for improved milk production in lactating cows has come with the price of increased early embryo loss, longer calving intervals, and a high frequency of services per pregnancy^1^. In feed-based systems, the cow’s dietary intake is more often than not outmatched by the biological processes involved post-parturition, mainly milk production and uterine repair. Cows experience fat mobilization, ketosis, and a surge in hormone biosynthesis, which ultimately changes their metabolic status^2^. Non-esterified fatty acids (NEFAs) and ketone bodies such as β-hydroxybutyrate (BHB) are elevated during the postpartum (pp) period and these have been associated with an increased incidence of endometritis^3^, making them interesting indicators of pregnancy outcome. Even though their production level peaks around week 2 pp^4^, blood serum levels can be detected well into the pp period and remain indicative of a predisposition for ketosis and periparturition diseases^5,6^. These metabolites appear to cross the blood-follicle barrier in ovaries, as they have been enriched in follicular fluid of developing follicle^7^. As the bovine ovarian cycle is resumed around day 10 postpartum (dpp), the oocytes growing within pre-antral follicles are exposed to elevated circulating metabolites in the follicular environment^8^. From 60 to 80 days, the oocyte is not only growing in size but also accumulating the necessary gene transcripts, proteins, and epigenetic marks necessary to sustain embryo survival^9^.

While no significant morphological and phenotypical changes were observed in oocytes derived from lactating cows pre- and post-day 42^10^, various studies have described the dynamic methylation changes occurring during oocyte maturation and fertilization. Overall methylation levels in fully matured oocytes were higher than their germinal vesicle stage counterparts^11^ and extensive demethylation in fertilized oocytes is expected to occur prior to embryonic genome activation^12^. Moreover, imprinted genes in oocytes are hypermethylated depending on their time of collection pp^13^. *In vitro* studies have also shown that concentrations of fatty acids similar to what is experienced pp in lactating dairy cows impair maturation, fertilization, cleavage, and blastocyst formation rates^7^. Using *in vitro* approaches, we have shown how maturation conditions impact methylation marks in embryos prior to embryonic genome activation^14,15^. These data support the hypothesis that the homeorhetic metabolism of lactating cows impacts the methylation status of oocytes, ultimately impairing their developmental ability. Here we describe, for the first time, the genome-wide methylation profile of *in vivo* oocytes collected from cows experiencing a negative energy balance, using whole-genome bisulfite sequencing. This holistic approach should provide new insights into the epigenetic regulatory mechanisms governing the oocyte developmental competence of pp lactating cows.

## Results

### Early-postpartum (Epp) cows are metabolically divergent from mid-postpartum (Mpp) and cyclic heifers (CHs)

In order to assess the association of divergent metabolic status with the epigenome of oocytes in lactating cows throughout pp, initial metabolic profiling of multiparous cows was performed. Thirty cows were followed during the first 15 weeks of calving to assess their energy status. First, the weight of the cows was measured to exclude those gaining weight. Accordingly, a single cow started to gain weight in its first week pp, which is associated with low milk production, and was excluded from the experiment. We then profiled cows based on their energy balance status calculated from farm data and blood metabolite analysis of both NEFA and BHB according to thresholds obtained from previous studies^5,6^ linking these concentrations with mild negative energy balances. When collection points met 2 out of these 3 criteria (negative energy balance, NEFA or BHB above threshold), the sample was assigned as being collected during negative energy balance, and when 2/3 did not meet the criteria (positive energy balance, NEFA or BHB under threshold), the samples were considered to be reflective of a positive energy balance. After this selection, 11 cows met the negative energy balance criterium in early-postpartum (Epp; average dpp at collection: 37.1 ± 1.4 dpp; average parity: 2.5 ± 0.41), and 7 animals met the positive energy balance selection criterium during mid-postpartum (Mpp; average dpp at collection: 65 ± 1.5 dpp; average parity: 3.0 ± 0.49). These cows reached their weight nadir around 37.6 ± 3.9 dpp and lost, on average, 50.4 ± 4.4 kg from the beginning of parturition, averaging a loss of 1.5 ± 0.18 kg/day.

In addition, these cows produced an average milk yield of 37.7 ± 1.5 kg/day, with a concurrent negative energy balance average of −25.5 ± 5.0 MJ/day prior to oocyte collection. During the collection of Epp oocytes, cows maintained a negative energy balance average (−19.75 ± 3.5 MJ/day). Interestingly, Mpp cows still exhibited a negative energy balance on average, although these were very close to a positive energy balance with a standard error of the mean (SEM) overlapping with positive energy balance values (−0.76 ± 3.15). Epp cows still had a significantly lower energy balance at the time of oocyte collection compared with Mpp cows (p < 0.001), indicating that these cows experienced early lactation energy balance deficits and recuperated toward a positive energy balance at later stages of lactation.

**Figure 1:**
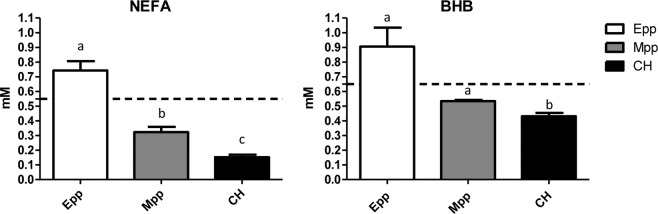
Blood metabolite concentrations [mM] of NEFA and BHB from cows selected during early postpartum (Epp), mid postpartum (Mpp) and heifers (CH). Dotted lines represent threshold of NEB found in the literature (NEFA = 0.55 mM Fenwick, 2008; BHB = 0.65 mM Girard, 2015), where letters show significant differences between groups and p < 0.05). Error bars represent the standard error of the mean.

Blood metabolite analysis revealed similar patterns, with average levels of NEFA above the threshold^5^ in Epp samples and significantly different than the levels found in the serum of Mpp and cyclic heifers (CHs) (Fig. 1, p < 0.05). The same pattern was found in BHB levels, where Epp BHB serum levels averaged above the threshold and Mpp BHB serum levels were, on average, under the threshold and closer to the CHs. Animals selected for early lactation negative energy balance, on average, exhibited significantly higher levels of both NEFA and BHB (Fig. 1, p < 0.05) compared with the Mpp and CH groups, whereas Mpp animals only had NEFA levels significantly higher than the CHs (p < 0.05). Blood metabolite analysis during the ovum pick-up procedures revealed that CHs were not metabolically challenged, and their metabolite concentrations were significantly different from the Epp negative energy balance animals but not significantly different than the Mpp animals (Fig. 1). These results indicate that, while not all cows experience a negative energy balance, selection of cows based on body weight loss as well as energy balance reveal oocytes collected at metabolically divergent states during pp, with Mpp animals resembling metabolically non-challenged CHs. Epp cows experienced a significantly lower energy balance as well as increased levels of circulating blood NEFA and BHB metabolites than Mpp cows and CHs.

### The epigenetic landscape of Epp oocyte genomic features diverges from Mpp and heifer oocytes

Following the different metabolic profiles of the Epp, Mpp, and CH groups, three pools of oocytes from each condition were submitted for genome-wide methylation profiling. Unique read alignments varied from 128,895,787 to 254,778,605 reads and the corresponding deduplicated CpG coverage at 1x was from 49.7% to 89.9%, with a coverage depth ranging from 1.0x to 4.7x per replicate (Supplementary Table S11). Global CpG methylation percentages were 61.3 ± 4.6%, 68.9 ± 1.0%, and 69.2 ± 0.1% in the Epp, Mpp, and CH groups, respectively. Concerning the methylation of genomic features (Table 1), there was consistency in the level of methylation across features, with similar methylation patterns across all groups. Epp oocytes had lower methylation levels for all genomic features compared with the Mpp and CH groups. Genomic feature methylation levels of CpG islands, promoters, and transcriptional units (TUs) were lower than global CpG methylation levels across the genome in all groups (Table 1). As such, high global CpG methylation averages seem to be caused by the high methylation levels of repetitive elements (e.g., LINE, SINE, LTRs) (Table 1).

**Table 1:** Methylation percentages of genome features in bovine oocytes derived from early postpartum (Epp), mid postpartum (Mpp) cows and cyclic heifers (CH).

Genomic features	Methylation (%)		
	Epp	Lpp	CH
CpG Islands	26.1	30.2	29.9
Promoters	36.5	41.9	41.6
Transcriptional units	34.4	41.1	40.9
Gene bodies	61.0	64.8	64.7
Intergenic	52.2	58.9	58.5
LINE1	67.8	74.1	73.7
LTR	63.1	71.2	70.7
SINE	67.7	78.4	77.8
Global	61.3	68.9	69.2

**Figure 2:**
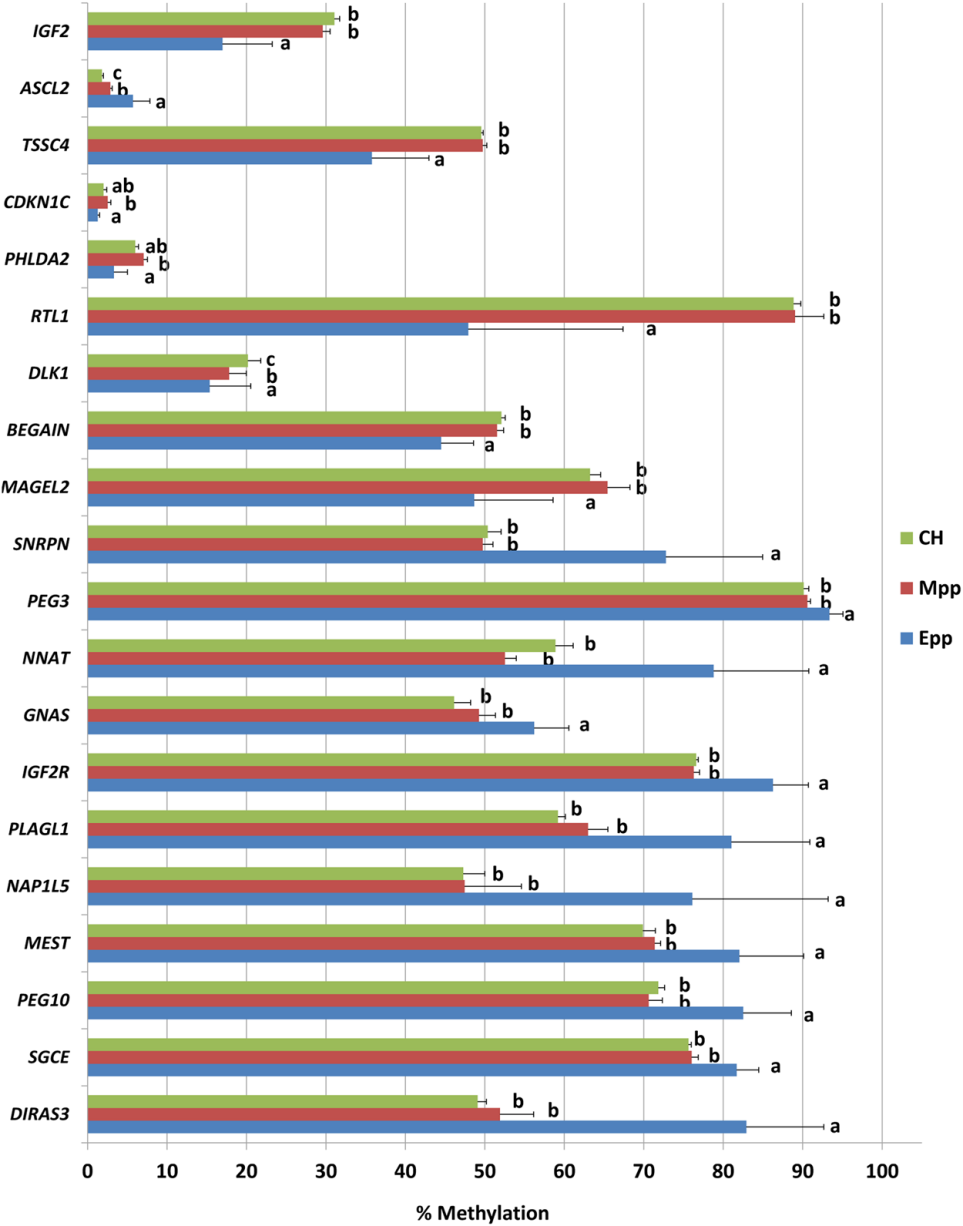
Methylation levels of genomic imprints gene bodies from early-postpartum (Epp), mid-postpartum (Mpp) and cyclic heifers (CH). Letters represent significance between groups where p < 0.05.

Although the overall methylation levels for entire gene bodies in the Epp group was, on average, lower than the Mpp and CH groups, the gene bodies of imprinted genes had a higher methylation status in most of the genes compared with their metabolically healthy counterparts. Epp oocyte methylation levels for gene bodies of imprinted genes were significantly different from 20 and 18 bovine imprinted genes in the Mpp and CH groups, respectively (Fig. 2, p < 0.05). Interestingly, only 2 Mpp genomic imprints differed from the CH oocytes, which could indicate that, coupled with the global and genomic feature methylation level distribution, the methylation profile of Mpp oocytes resembles the heifer epigenetic landscape.

**Figure 3:**
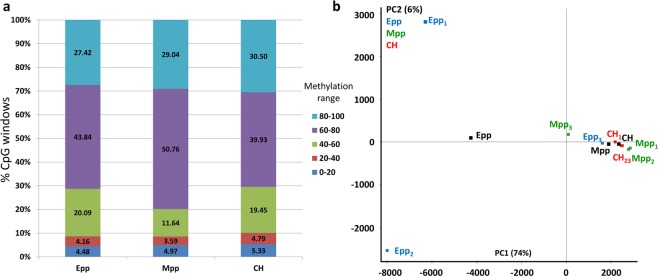
Methylation level distribution across the genome across samples. (**a**) Methylation levels of 150 CpG probes across the genome of the three groups of oocytes (Epp, Mpp, and CH). (**b**) Principal component analysis of the DNA methylation distribution in early-pp (Epp, blue), mid-pp (Mpp, green), and cyclic heifers (CH, red) with their corresponding averages (black).

**Figure 4:**
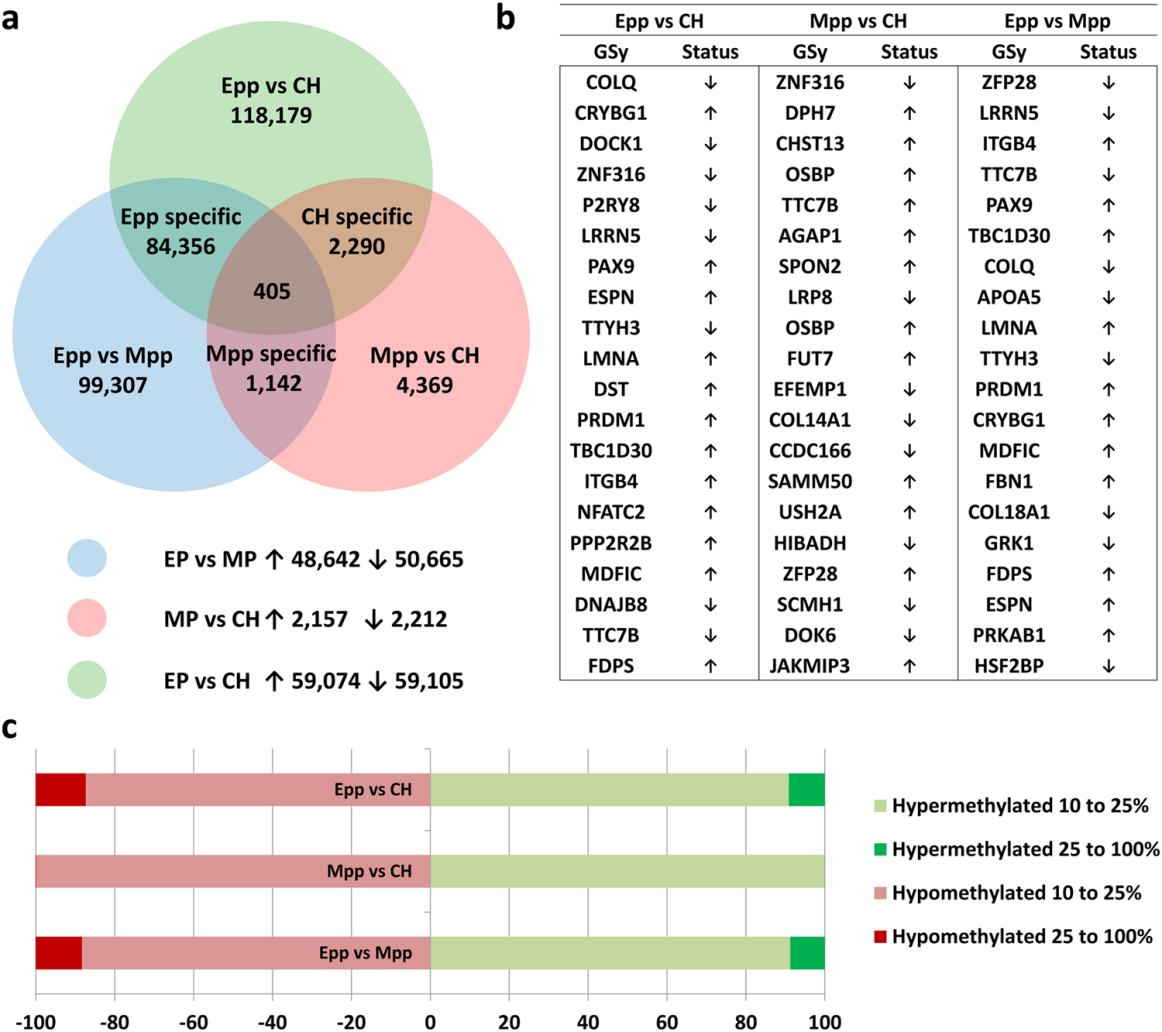
DNA methylation analysis of oocytes derived from early-postpartum (Epp), late postpartum (Lpp), and cyclic heifers. (**a**) Venn diagram of DMRs obtained by logistic regression (10% methylation difference, adjusted p < 0.05), with number of hyper- and hypo-methylated DMRs in each comparison, where 405 DMRs were found to be shared across all conditions (center). (**b**) Top 20 differentially methylated regions overlapping gene (GSy = gene symbol) body regions and their methylation changes in all three comparisons. (**c**) Fold methylation changes found in hyper- (right) and hypo-methylated (left) DMRs from pairwise comparisons.

As the whole gene body may be too large for the adequate characterization of DMRs, a more detailed analysis was performed by binning the genome into 150 CpG windows. Of the 357,863 windows created from the 9 datasets, 98.7% of these windows were quantified across all datasets. An overall representation of the methylation levels of these windows and the genome view of the respective replicates is shown in Fig. 3A, of which a slightly greater representation of 60 to 80% methylation was observed in the distribution of methylation windows for the Mpp samples. From these windows, differentially methylated regions (DMRs) were identified by logistic regression replicate testing with a >10% methylation change between conditions and corrected using the Benjamini-Horchberg method at p < 0.05. Principle component analysis (PCA)-based clustering of these windows is shown in Fig. 3B, with a high degree of clustering in the Mpp and CH but not Epp samples, which may be explained by the variable coverage depth compared with the Mpp and CH samples (Supplementary Table S11). Nevertheless, match distribution normalization and logistic regression filtering of the data still listed a high degree of DMRs found in the by comparing Epp with CH groups and Epp with Mpp groups (Fig. 4A). Additionally, the Epp group had greater methylation changes, with approximately 10% of its DMRs being between 25% and 100% of the methylation changes in both hypermethylated and hypomethylated DMRs (Fig. 4C). Pairwise comparisons between conditions revealed that DMRs overlapping gene body regions in all comparisons and the top 20 most differentially methylated regions from each comparison are listed in Fig. 4B (Supplementary Table S2–4). Additionally, lists of DMRs from each pairwise comparison were analyzed for gene ontology pathway enrichment with clusters of genes involved in metabolic pathways, hormone signaling pathways, and cellular structure pathways (Supplementary Table S5–7).

Pairwise comparison lists of DMRs were combined to identify changes exclusive to each biological condition. A total of 2,290 DMRs were identified as exclusive to the CH group compared with the Epp and Mpp groups, of which 1,014 and 1,183 were found to be hyper- and hypomethylated in CH compared with Epp and Mpp, respectively, despite their divergent metabolic profiles (Fig. 4A). Of these DMRs, 705 CH-specific DMRs overlapped with gene bodies. This proportion of DMRs in gene regions seemed higher than what was found initially in the genome (gene bodies representing 27.04% of windows in the genome) but this increase was also found in DMRs from all biological conditions (Supplementary Table 1). A notable increase was found in the proportion of DMRs overlapping CpG islands, where Epp-specific DMRs had 1.99-fold increased representation. This overrepresentation was 3.08-fold (Supplementary Table 1) when looking into the 405 DMRs that were shared across all groups (Fig. 4A), indicating that a proportion of methylation changes observed across all groups overlapped with CpG island regions of the genome. As for the Mpp-specific DMRs, 1,142 DMRs were found, which was the smallest variation of the three conditions. The bulk of the changes observed in DMRs stem from Epp-specific DMRs, totaling 84,356 DMRs. Of these, 32,990 overlapped with gene regions, corresponding to 39.1% of the genome features overlapping gene bodies, a 12.1% increase from the normal representation of this feature (Supplementary Table 1). Consistent with the dispersed PCA clustering of the replicates, Epp oocytes experienced more DNA methylation changes than their Mpp counterparts.

#### Global gene body methylation differences exclusive to Epp oocytes are involved in metabolic processes

To gain insight into the functional relevance of these changes, DMRs that overlapped with gene regions were selected for enrichment pathway and gene ontology analysis. When looking at Mpp oocytes from recuperating cows, a list of 265 DMRs overlapping genes showed low enrichment in biological processes such as immune response, down-regulation of DNA binding transcription, fatty acid oxidation and biosynthetic processes (Supplementary Fig. 1). These pathways were linked by the presence of phosphoinositide-dependent kinase-1 (*PDPK1*), serine/threonine-protein kinase D2 (*PRKD2*), protein kinase C beta type (PRKCB), TNF receptor-associated factor 2 (*TRAF2*), and NCK-interacting protein kinase (*TNIK*) genes, which are involved in the immune response and fatty acid processing (Supplementary Table S9). The low enrichment of DMRs over the number of putative target genes could indicate their small variation in the biological processes occurring in Mpp oocytes. On the other hand, CH-specific DMRs yielded a list of enriched KEGG pathways, with 517 genes involved in actin skeleton regulation, focal adhesion, and adherens junctions, as well as the Ras and Hippo signaling pathways. Genes connecting these pathways included serine/threonine-protein kinase PAK 3 (*PAK3*), integrin alpha-IIb (*ITGA2B*), integrin beta-8 (*ITGB8*), and epidermal growth factor receptor (*EGFR*) (Supplementary Table S10).

**Figure 5:**
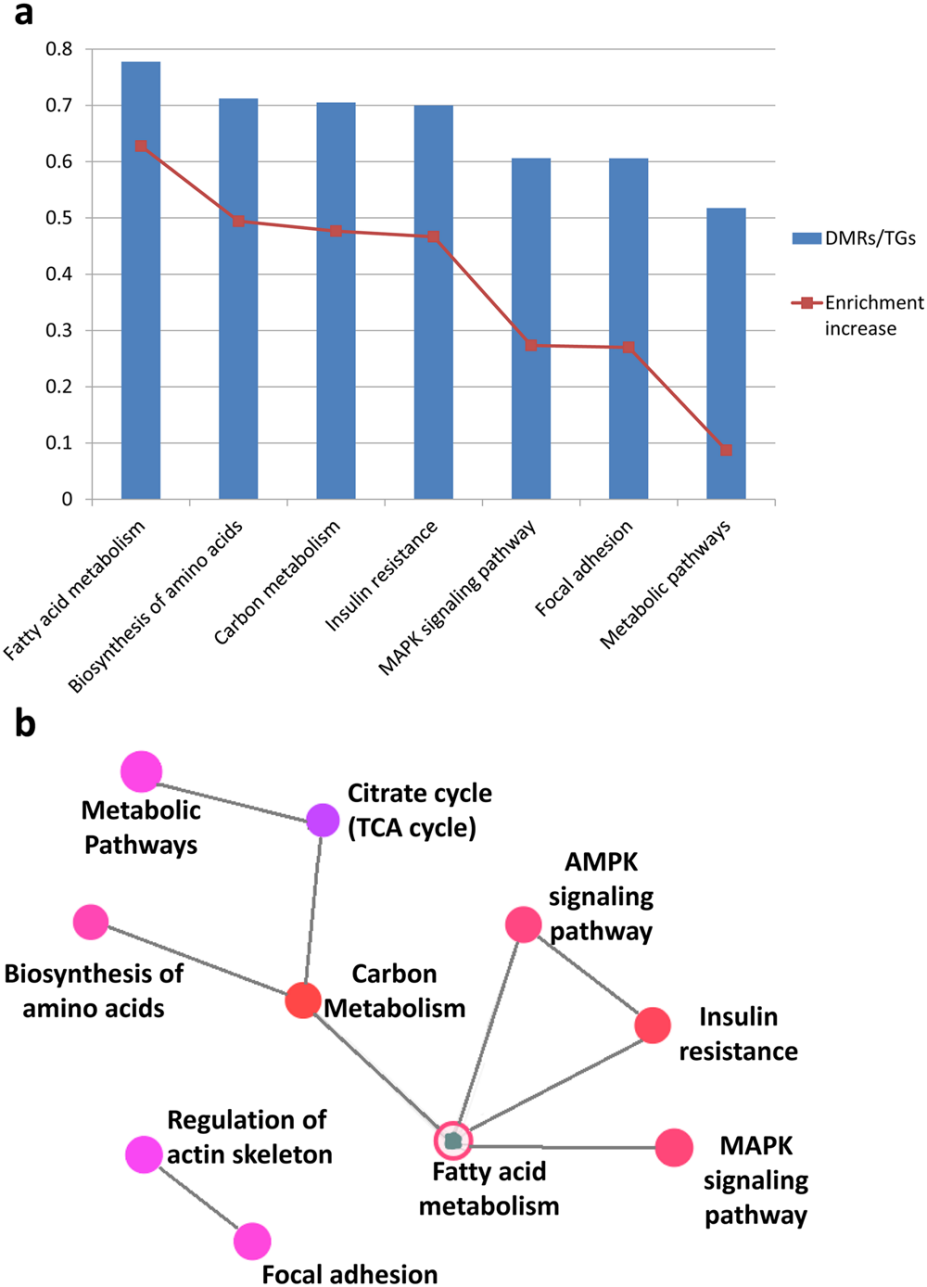
Functional relevance of early postpartum (Epp)-specific DMRs. (**a**) Top pathways from the KEGG pathway database involved with the DMRs found in the Epp group with their pathway enrichment increase. (**b**) KEGG database pathway network association with DMRs from the Epp groups and their interaction. DMRs/TGs: Ratio of differentially methylated regions over the number of genes targeted by the pathway. Pathways shown have adjusted p-values < 0.05.

As for the DMRs specific to Epp oocytes, a total of 10,114 genes were involved spanning multiple pathways, most notably enriched in metabolic pathways (761 genes), carbon metabolism (79 genes), fatty acid metabolism (42 genes), and amino acid biosynthesis (52 genes) (Fig. 5). Structural genes involved in focal adhesion and actin skeleton were also found (p < 0.05, false discovery rate (FDR) < 0.004, Supplementary Table S8). The low amount of DMRs specific to Mpp and CH oocytes, as well as the low degree of gene enrichment in biological processes and pathways with a higher FDR (p < 0.05, FDR > 0.05) compared with the high number of DMRs. The enrichment pattern in Epp oocytes indicates that the metabolic stress experienced during early lactation may confer high variation to the methylome, possibly impairing their metabolism and structure.

#### DMRs exclusive to Epp metabolically stressed oocytes are found in gene bodies of genomic imprints

**Figure 6:**
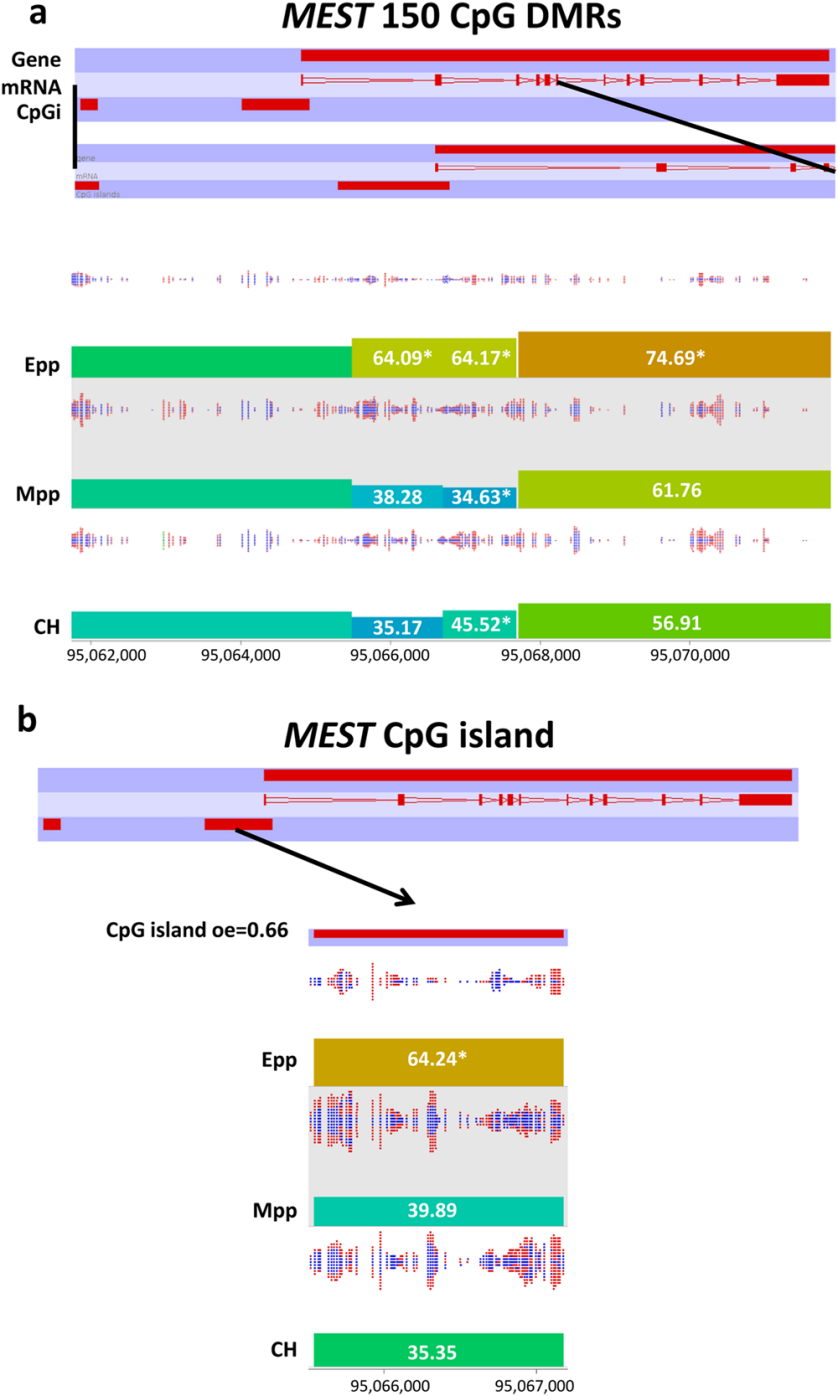
DMRs around the MEST locus. (**a**) 150 CpG windows DMRs overlapping the *MEST* gene body region. (**b**) Methylation quantification of the CpG island overlapping the MEST gene body. Methylation percentages are found in the bars, where * represents significant (adjusted p < 0.05) methylation differences either in the early-postpartum values or across all conditions.

Since genomic imprints have an impact on the ultimate developmental competence of the offspring, with improper imprinting status being associated with disease, we identified DMRs overlapping the genes of selected imprints (Fig. 2). Only one DMR was found across all conditions, overlapping the *MEST* gene, upstream of the coding sequence (Fig. 6). This DMR also overlapped a CpG island region, for which separate quantitation revealed it to also be differentially methylated in Epp oocytes but not in Mpp and CH oocytes. 48 additional neighboring DMRs were differentially methylated only in Epp oocytes (Table 2) with a majority of these being hypermethylated compared with the Mpp and CH groups, 25 DMRs overlapped CpG islands, and 33 DMRs overlapped exons of those genes, indicating that these neighboring CpG islands and gene body regions are sensitive to metabolic stress. Interestingly, a cluster of 7 adjacent Epp-specific DMRs was hypermethylated compared with CH and Mpp oocytes with 6 of these DMRs overlapping two CpG-rich regions inside of the *GNAS* gene body, spanning 11.8 kbp (Table 2). Taken together, the methylation variations observed in the gene bodies of imprinted genes in Epp oocytes present an interesting landscape to investigate the impact of metabolic stress on the developmental competence of oocytes.

**Table 2:** List of early postpartum oocyte-specific DMRs overlapping imprinted gene bodies.

Gene	Probe location	Methylation level (%)			Overlapping CpG island	Overlapping exon
		Epp	Mpp	CH		
*DIRAS3*	Chr3:77572944–77582220	68.91	54.76	54.79	+	+
*SGCE/PEG10*	Chr4:11911539–11912583	67.52	35.40	33.34	+	+
*MEST*	Chr4:95066705–95067686	64.18^*^	34.63^*^	45.52^*^	+	+
*MEST*	Chr4:95067710–95072935	74.69	61.76	56.91	−	+
*NAP1L5*	Chr6:37509075–37510923	71.47	45.96	43.66	−	+
*IGF2R*	Chr9:97639143–97641473	74.90	84.92	89.40	−	−
*IGF2R*	Chr9:97654566–97658868	83.48	71.02	68.94	−	+
*IGF2R*	Chr9:97658884–97661928	68.46	40.84	39.89	−	−
*IGF2R*	Chr9:97664415–97669428	79.96	56.80	54.21	+	+
*IGF2R*	Chr9:97669459–97672435	76.73	58.01	55.43	−	−
*IGF2R*	Chr9:97677102–97678968	75.23	50.88	47.24	−	−
*IGF2R*	Chr9:97683111–97685609	82.86	62.47	68.34	−	+
*IGF2R*	Chr9:97696872–97698360	84.14	67.99	64.95	−	+
*IGF2R*	Chr9:97698361–97700386	82.20	68.84	64.51	−	−
*IGF2R*	Chr9:97723665–97725285	88.60	76.54	75.85	−	+
*IGF2R*	Chr9:97729941–97731789	85.35	75.30	71.92	−	−
*IGF2R*	Chr9:97731832–97733485	73.48	52.29	51.05	+	+
*GNAS*	Chr13:58017810–58022193	60.92	49.16	50.34	−	+
*GNAS*	Chr13:58028217–58033182	73.78	41.85	42.32	+	+
*GNAS*	Chr13:58033183–58034298	69.10	39.73	34.80	+	−
*GNAS*	Chr13:58034299–58035180	70.20	46.54	41.68	+	−
*GNAS*	Chr13:58035201–58036679	73.48	55.78	49.28	+	−
*GNAS*	Chr13:58036729–58037912	63.27	34.61	28.17	+	−
*GNAS*	Chr13:58037929–58040073	76.55	46.12	45.39	+	−
*GNAS*	Chr13:58040227–58046005	64.63	53.93	52.90	−	−
*GNAS*	Chr13:58047147–58048143	19.79	37.44	30.89	+	+
*NNAT*	Chr13:67118471–67119734	77.60	40.88	46.39	−	+
*NNAT*	Chr13:67119735–67122534	73.18	60.18	60.95	−	+
*SNRPN*	Chr21:13703–24837	70.60	37.27	44.83	−	+
*SNRPN*	Chr21:24838–26023	66.22	34.92	32.17	+	+
*MAGEL2*	Chr21:784775–797175	47.18	31.21	27.70	−	+
*BEGAIN*	Chr21:67073406–67074663	53.60	76.84	85.63	+	+
*BEGAIN*	Chr21:67074664–67076413	50.29	67.12	68.79	−	+
*BEGAIN*	Chr21:67076414–67079286	41.83	66.02	63.67	−	+
*BEGAIN*	Chr21:67082421–67084236	50.61	65.72	72.34	−	+
*BEGAIN*	Chr21:67084286–67085973	39.77	53.52	54.18	+	−
*BEGAIN*	Chr21:67089429–67092594	77.28	61.97	60.42	−	−
*BEGAIN*	Chr21:67092595–67095173	64.69	29.43	24.94	+	−
*BEGAIN*	Chr21:67095174–67098689	61.99	22.32	22.41	+	−
*BEGAIN*	Chr21:67098706–67099431	53.50	4.28	3.32	+	+
*RTL1*	Chr21:67426306–67427887	49.26	61.78	67.91	+	+
*RTL1*	Chr21:67427910–67429035	66.64	94.10	91.23	+	+
*RTL1*	Chr21:67429048–67430254	54.79	90.02	89.27	+	+
*RTL1*	Chr21:67430264–67432092	65.54	82.26	88.41	+	+
*TSSC4*	Chr29:49836440–49837857	74.90	87.95	87.48	−	+
*ASCL2*	Chr29:49957329–49959108	60.98	46.72	45.26	+	+
*IGF2*	Chr29:50045707–50047790	28.20	39.23	39.61	−	+
*IGF2*	Chr29:50061341–50062955	43.98	61.46	64.07	+	+
*IGF2*	Chr29:50064682–50065982	43.86	80.23	80.77	+	+

^*^Denotes a single DMR differentially methylated in all conditions. + and − denote presence or absence of overlap, respectively.

## Discussion

The follicular milieu of the oocyte is crucial for its proper maturation and the acquisition of epigenetic marks. These will dictate its ability to sustain fertilization and develop into viable offspring, and altering this profile during *in vitro* maturation can reduce developmental capabilities^13,16^. As such, lactating cows have been previously described as exhibiting an alternate metabolic profile associated with phenotypic changes regarding folliculogenesis and ovulation, ultimately resulting in poor reproductive quality^17^. To the best of our knowledge, this study is the first to demonstrate that cows experiencing a negative energy balance exhibit differentially methylated regions in genes involved in metabolism and development. To gain molecular insight into the impact of lactation-induced stress during pp on the gamete population during follicular waves, we profiled cows undergoing a negative energy balance both physiologically and metabolically and evaluated the epigenetic landscape of the subordinate oocyte population during (week 5–6 pp) and exiting (week 9–10 pp) an exposure to metabolic stress. Physiologically, we obtained oocytes at a time when the cow has resumed its ovarian cycle, even though estrous signals are somehow silent^18^, and recuperated from pp ailments, like uterine involution and body weight loss, and is ready to be inseminated for reproduction^19^. Using nulliparous heifer oocytes, we were able to characterize what defines subordinate oocytes grown in unchallenged metabolic conditions and compare epigenetic patterns with oocytes exposed to metabolic stress.

Indeed, 97% of cows experienced a weight loss prior to oocyte collection, with only one cow gaining weight immediately pp that produced negligible amounts of milk and was excluded from further analysis. Therefore, most of the cows in the present study behaved consistently with a negative energy balance as it has been described in other studies^4,20^. Furthermore, cows were selected based on additional criteria including energy balance and metabolic level. A previous report found that cows return to positive energy balance around 41 days pp^21^. Interestingly, cows selected for the present study still experienced a negative energy balance around this time, but also exhibited overall negative energy balance at the time of Mpp (week 9–10 pp). Even if cows exhibited an average negative energy balance, their metabolic levels had significantly reduced to being close to the levels of unchallenged heifers. This suggests that, though Mpp cows return to metabolically basal NEFA and BHB concentrations, the animals used in this study were still physiologically challenged. A previous study described that cows exit the metabolically challenging period at d42 based on body condition score increase, while NEFA and BHB values return to normal levels preparturition, with peak production of these metabolites occurring around week 2 pp^9^. However, body weight, insulin and glucose levels in these cows remained low even after d42, suggesting that these could be more accurate indicators of energy balance, which would correspond to the energy balance averages of the cows included in this study. Nevertheless, we found significantly different averages for energy balance, NEFA, and BHB, indicating that cows do recuperate from negative energy balances during lactation, although perhaps at a more delayed rate than suggested in the literature.

Our study revealed that the Epp period has a stronger metabolic impact on cows than Mpp time points, which metabolically resembled CHs. We subsequently set out to characterize the epigenetic impact of this metabolic stress on oocytes. The methylation profiles of pools of Epp oocytes were highly heterogeneous compared with Mpp and CH oocytes, indicating the high impact variability of early lactation on oocytes’ methylation status. The animal- and follicle-specific effects of metabolic stress on the oocyte epigenome cannot be ruled out. Future research on individual cow- and follicle-specific sensitivities to stress will explain the basis for such variation in response to metabolic state. Nevertheless, average quantification of genomic features exhibited similar differences in Epp oocytes compared with Mpp and CH oocytes, with global methylation levels being lower in Epp samples and differences between features being conserved across all biological conditions. Interestingly, methylation levels in CpG islands were overall lower than in other features, as has been previously observed in bovine oocytes^22^. A similar methylation level in CpG islands and differential clustering within certain conditions have also been described from the whole-genome bisulfite sequencing of pig embryos, although levels of global methylation between oocytes and embryos are not comparable, as the latter undergoes extensive demethylation^23^. This study showed ratios of methylation in gene features spread similarly across blastocyst groups, indicating a similar organization of the genome in all conditions. In the present study, the overall organization of genomic feature methylation of Epp oocytes was conserved compared with Mpp and CH oocytes, with slight hypomethylation in both comparisons. Interestingly, we report higher oocyte methylation levels, as well as lower non-CpG methylation levels, than previously described in bovine oocytes^11,22^ using both reduced representation bisulfite sequencing (RRBS) and whole genome bisulfite sequencing (WGBS) techniques, which have been reported to generate different average methylation levels^24^. Additionally, both CpG and non-CpG methylation levels in somatic cells (e.g., fibroblasts) have been reported to be lower^25^ than oocytes^26^. While some of our oocytes exhibited lower non-CpG levels (<3%, Supp. Table S11), the levels of overall methylation seem to hover between the low levels reported in oocytes and the high levels reported in somatic cells^11,22,26^. Nevertheless, global oocyte methylation levels has been reported to be around 60%^27^ in humans. Additionally, when investigating oocyte-specific DMRs such as exon 10 of insulin growth factor 2 (*IGF2*; chr29: 50062057–50062476), methylation levels were consistent with published reports^28,29^. This region is completely methylated in sperm (96.04%) and partially methylated in oocytes depending on the stage and maturity of the oocyte, ranging from 28.9% in mature oocytes from large follicles^28^ to 73.7% in primordial follicle oocytes^29^. Epp oocytes had a methylation level of 33.2% compared with 72.1% and 77.2% in Mpp and CH oocytes, respectively. This variation could be explained by the variation in follicle size and oocyte stage, which were pooled in this analysis. Inversely, KvDMR1, a regulating region of the cyclin-dependent kinase inhibitor 1 C gene (*CDNK1C*; chr29:49554319–49554666), is methylated at high levels in human oocytes and low levels in sperm, although aberrant patterns have also been reported^30^. Similar levels of allele-specific methylation were found in bovine somatic tissues^31^. In the present study, only Epp oocytes were hypermethylated with an average methylation level of 81.0% compared with 48.8% and 59.0% in Mpp and CH oocytes, respectively. While somatic cell contamination cannot be excluded, regions described in this study behaved similarly to previously described regulatory regions^28–31^.

Upon closer inspection, compartmentalizing the genome in fixed CpG windows revealed specific regions that were differentially methylated. Subsequent clustering analysis revealed a higher level of variation in DMRs specific to Epp oocytes. Functionally, gene ontology revealed that fatty acid metabolism, oxidation, and degradation were involved in Epp, Mpp, and CH oocytes, respectively. A previous report outlined the differential fatty acid uptake during early embryo cleavage, with embryos failing to develop beyond the 4-cell stage having significantly higher concentrations of saturated fatty acids^32^. Coupled with previous reports of developmental failure in oocytes matured with high NEFA concentrations, the fatty acid oxidation and degradation found in Mpp and CH oocytes could suggest a metabolic direction of fatty acid metabolism that favors developmental competence. Similarly, BHB acts as a histone deacetylase inhibitor in mice, but not in cows, although it has been shown to modulate lipid metabolism genes in bovine somatic cells^33,34^. Similarly, we suggest that exposure to such metabolites during Epp has an impact on the methylation of genes involved in cellular lipid metabolism. In Epp-specific DMRs, 18 genes were found to be differentially methylated and involved in at least 4 pathways with multiple genes being isozymes or similar in structure. Of these, *IDH3A* is involved in tricarboxylic acid cycle metabolism in the nuclei of early cleavage embryos prior to zygotic embryo activation^35^, suggestive of a role in early developmental competence. Also, *PI3KCD* is involved in follicle growth, as *PIK3CD* null mice are subfertile, with fewer growing follicles and a dampened response to gonadotropin stimulation^36^. DMRs were also found in genes involved in the cross-talk between bovine oocytes and surrounding cumulus cells including *ACO1* and *ACO2*, both involved in carbon metabolism and over-expressed in oocytes co-cultured with cumulus cells^37^. Also, isoforms of protein kinase B (*AKT2/3*) were differentially methylated in Epp oocytes, which is known to impact meiosis through the organization of microtubules in mice, subsequently influencing fertilization outcomes^38^.

DMRs specific to Mpp oocytes were also involved in various reproductive processes. Of these, protein kinase C (*PKC*) was previously implicated in improving maturation, where stimulation of *PKC* resulted in increased pronuclear formation and faster meiotic resumption rates in bovine oocytes^39^. Another protein kinase, 3-phosphoinositide-dependent protein kinase-1 (*PDK1*), is crucial for primordial follicle survival in mice, where PDK1 knockout oocytes cause infertility and premature ovarian aging^40^. Taken with the remaining DMRs from Mpp oocytes, the results from this work demonstrated the effect of metabolic stress on gene regulatory mechanisms, explaining the failure associated with embryo loss at first service post-calving. Additionally, CH-specific DMRs might provide clues regarding the methylome of oocytes under standard conditions that can easily be associated with developmental competence. Genes such as integrin beta 8 were downregulated during the *in vitro* maturation of porcine oocytes, although its influence on developmental competence remains unclear^41^. Lysophosphatidic acid receptor (*LPAR1*) might modulate cumulus oocyte complex quality, as supplementation with LPA during maturation improved the expression of the oocyte quality markers follistatin and growth differentiation factor 9 (*GDF-9*), although d7 blastocyst rates did not improve^42^. Moreover, epidermal growth factor receptor (*EGFR*) and *FYN* kinase play a role in the completion of meiosis^43,44^. Although gene expression validation studies are needed to further confirm the scale and direction of these methylation differences, our results indicate that the metabolic status of post-calving cows may significantly influence the activity of genes associated with the developmental competence of oocytes.

Previous reports have linked postpartum NEFA concentration with differential imprint acquisition in pp oocytes^12^, where varying methylation levels were screened in imprinted genes along their gene bodies, notably *MEST*, *IGF2R*, and *SNRPN*. Although not at the same positions, we report specific DMRs found in the gene bodies of these imprints and others, indicating early lactation metabolic stress had a similar impact on imprint acquisition pp. Similarly, *MEST*, a gene expressed from the paternal alleles in the mesoderm and its differentiated lineages, is dynamically methylated in mice in the upstream promoter region of a CpG island similar to the one we observed^45^. Usually fully methylated in the maternal germline, *MEST* was differentially methylated in fully grown and freshly ovulated oocytes, while hypermethylated in oocytes cultured *in vitro*, highlighting the dynamic nature of imprint acquisition and its sensitivity to different growth conditions. Inverse to overall global methylation levels, DMRs overlapping imprinted gene bodies had higher methylation levels during Epp compared with late postpartum and heifers in 28 of the 34 DMRs listed. Additionally, numerous studies report a positive correlation between transcribed gene body methylation and expression level, indicating that the hypermethylation of gene bodies could be associated with upregulated expression^46–48^. This suggests that imprints are generally hypermethylated in Epp oocytes, modulating their expression regardless of putative imprint status. Further validation with gene expression analysis is needed to confirm the functional relevance of the epigenetic modification of these regions.

In conclusion, here we describe the genome wide methylation profiles of oocytes retrieved from cows under metabolically divergent conditions, from which we identified relevant biological processes that could be involved in the reproductive failure of lactating cows. We demonstrated that, while metabolically recuperated, cows do exhibit a negative energy balance Mpp, though differential methylation was more pronounced in oocytes from Epp cows. Nevertheless, the aforementioned approach provided an array of new DMRs in the bovine genome to investigate the relationship between metabolic stress and developmental capacity. Future studies will aim to validate the correlation between gene body methylation and gene expression to establish how these genes are involved in the developmental failure associated with Epp metabolic stress.

## Materials and Methods

### Animal handling, oocyte and blood collection from lactating pp cows and CHs

Animal handling was carried out in accordance with the 2015 German Law of Protection (TierSchG & TierSchVersV). Experimental protocols performed on cows in this study were approved by the state office for Nature, Environment, and Consumer Protection of North Rhine-Westphalia, Germany (Landesamt für Natur, Umwelt und Verbraucherschutz Nordrhein-Westfalen, Deutschland). The blood sample collection and ovum pick-up (OPU) procedures were approved under license numbers 84–02.04.2015.A139 and 84–02.04.2014.A500, respectively. Thirty Holstein Friesian cows and eight nulliparous cyclic heifers were used for this experiment. Lactating cows were monitored daily up to 15 weeks pp for body weight, feed and concentrate intake, milk yield and composition. Starting at week 5 pp, oocytes were collected from cows using transvaginal ultrasound-guided OPU. Using an ultrasound probe with a cannula connected to an aspiration pump, subordinate follicles sized 3–8 mm were aspirated. Once collected, the oocytes were mechanically denuded using hyaluronidase (1 mg/mL) and vigorous vortexing for 5 minutes. The denudation reaction was terminated by transferring the oocytes into 3 mL of TCM199 supplemented with bovine serum albumin (1 g/mL). Thereafter, every oocyte was checked for the presence of remaining cumulus cells under a stereomicroscope at high resolution by an experienced technician. In cases with remaining cumulus cells, individual oocytes were treated with a Stripper-pipette (EZ-Strip^TM^, Gynemed, Lensahn, Germany) until the remaining cumulus cells were fully removed. The oocytes were then carefully washed three times in PBS without calcium and magnesium supplemented with polyvinyl alcohol (0.3 mg/mL) and those that did not appear lysed were snap-frozen at −80 °C until further use. This procedure was repeated weekly until week 10 pp. This OPU procedure was also performed in 8 nulliparous heifers for 5 consecutive weeks. In parallel to OPU, 20 mL of blood was collected from each animal for blood metabolite analysis. The serum was separated and frozen at −80 °C for future analysis.

### Energy balance assessment of cows and oocyte selection pooling

Body weight curves for all cows were drawn to ensure a lack of weight gain in the Epp phase. Cows experiencing immediate weight gain pp were excluded from further analyses to ensure the selection of cows experiencing a negative energy balance post-calving exclusively. A weekly energy balance average was calculated to assess energy status using the following equation

1 $$EB=DMIe+Ce-BWe-MYe$$

where the energy balance (EB) is the result of dry matter intake energy (DMIe), plus the concentrate energy (Ce) fed to the cows, and minus the maintenance of body weight energy (BWe) and milk yield energy (MYe), which was calculated using milk weight and composition (fat, protein, and lactose percentage). Additionally, blood metabolite analysis was performed spectrophotometrically (HORIBA, Montpellier, France) for both β-OHB (Kit #RB1008, Randox Laboratories, Crumlin, United Kingdom) and NEFA (#434–91795, Wako Chemicals GmbH, Neuss, Germany) following a published protocol^49^. Serum NEFA and BHB values over the thresholds described in the literature^5,6^ were associated with a negative energy balance. Accordingly, 3 phenotypes were selected: oocytes from Epp, (w5–6 pp), oocytes from Mpp, (w9–10 pp), and oocytes from CHs as non-metabolically challenged controls. Oocytes were pooled into biological triplicates for each phenotype (Epp, n = 62 (20.7 ± 2.1 oocytes/replicate); Mpp, n = 64 (21.3 ± 0.6 oocytes/replicate); CH, n = 181 (60.3 ± 2.5 oocytes/replicate)) for bisulfite conversion.

### Oocyte DNA bisulfite conversion and isolation

Oocytes in each triplicate were lysed and bisulfite treated using the EZ-DNA Methylation-Direct Kit (Zymo Research, Freiburg, Germany) according to the manufacturer’s instructions with some modifications. Oocytes were initially digested in 10 µl of digestion buffer (Zymo Research) and 1 µl of proteinase K (Zymo Research), where digestion reactions were scaled according to the volume of the starting sample (up to 27 µL, with a 3-fold limit) at 50 °C for 20 minutes. The resulting digested samples were split according to the previous lysate volume into up to 3 reactions and were bisulfite converted using the provided CT conversion reagent (Zymo Research) at 98 °C for 8 minutes followed by a final incubation at 64 °C for 3.5 hours. These conversion reactions were bound to collection columns, desulphonated, and washed according to the manufacturer’s instructions. After this purification step, reaction replicates were eluted using 8 µL of the elution buffer provided in the Pico Methyl-Seq Library Prep Kit^TM^ (Zymo Research). The resulting bisulfite-treated DNA was used for library preparation.

### Library preparation for whole-genome bisulfite sequencing

Sequencing libraries were prepared from the resulting bisulfite-treated DNA from pooled oocytes using the Pico Methyl-Seq Library Prep Kit^TM^ (Zymo Research) according to manufacturer’s recommendations with some modifications. Briefly, bisulfite-treated DNA was pre-amplified with a primer concentration reduced to 20 µM to prevent primer-dimer formation in the final library. The DNA was then purified using a DNA binding buffer to sample ratio of 5 to 1 during all of the purification steps to increase fragment recovery. Following this purification step, the pre-amplified DNA was further amplified for 10 PCR cycles during the amplification step of the library preparation, according to the manufacturer’s recommendations. The resulting reaction was purified and further amplified with index primers. The resulting PCR product was purified and the fragment size and quality were assessed using the Agilent High Sensitivity DNA Assay with a Bioanalyzer (Agilent, Waldbronn, Germany). Library quantification was achieved by qPCR using the KAPA Biosystems^TM^ Library Quantification Kit (Roche, Mannheim, Germany). Libraries were single-end sequenced for 114 cycles on an Illumina HiSeq. 2500 using TruSeq v3 chemistry (Illumina GmbH, Munich, Germany). Raw sequencing data were demultiplexed according to the index primers added during library preparation, available on the European Nucleotide Archive (ArrayExpress accession number E-MTAB-8191), and used for further processing.

### Raw sequence data processing

Raw sequence data were subjected to quality control with FastQC v0.11.6^50^ and subsequently trimmed with Trimmomatic 0.36^51^ hard trimming of 10 bp on each end of the fragment to reduce the base composition bias of the reads, as per the aligner’s recommendations, with the following parameters: SE –threads 8 ILLUMINACLIP:TruSeq. 3-SE.fa:2:30:20 MAXINFO:83:1 LEADING:20 TRAILING:20 CROP:100 HEADCROP:10 MINLEN:20. Trimmed sequences were verified on FastQC and mapped to the bisulfite converted bovine genome generated from the reference bovine genome (Ensembl release 93) using the Bismark tool^52^. Trimmed sequences were aligned using the Bismark v0.19.0 tool with the Bowtie 2 aligner^53^ with the single-end read default aligning parameters. After alignment, duplicate sequences were removed and CpG methylation calling was extracted using the deduplication and methylation extraction modules of the Bismark tool. Methylation coverage output files were imported into SeqMonk v1.44.0^54^ for further analysis.

### DNA methylation quantitative analysis

For general quantitation of genome features between conditions, the methylation percentage for probes spanning genome features was calculated by multiplying the ratio of methylated counts to methylated and demethylated counts by 100. CpG islands, promoters, TU, LINE, SINE, and LTR probes were delimited using Ensembl release 93 genome annotation and methylation levels were calculated using the same approach. Bovine-specific genomic imprinted probes were selected using the gene imprint list^55^ as well as imprinted genes found in the literature^56,57^ using the Ensembl notation found in Seqmonk. Promoters were set to span 1,000 bp upstream of the whole gene body, where TUs covered 500 bp downstream of the annotated start of the gene body.

To ensure unbiased analyses, the genome was binned in 150 CpG windows using the read position probe generator and 1 minimum read count to include position and 150 valid positions were selected. Windows were generated across all replicates for a total of 357,863 windows in all 9 datasets. To enable maximum data retention, methylation percentage of the 150 CpG site-binned windows was calculated using the bisulfite methylation over features quantitation pipeline with a minimum count of one to include position and 20 minimum observations to include a feature described in the literature^23^. To enable logistic regression comparison of quantitated windows, a value filter was applied to every window with a value between 0 and 100 across all 9 datasets. When filtered, the resulting total number of windows was 353,294 and these windows were quantitated again using the bisulfite methylation over features pipeline. This quantitation was subsequently normalized across all data sets using the match distribution quantitation tool. Differentially methylated regions identified through logistic regression were submitted to gene ontology and KEGG pathway enrichment analysis using the network analyst^58^.

### Statistical analysis

When comparing energy balance status, data from biological conditions were compared using ANOVA, where a p < 0.05 was found to be statistically significant. Data are presented as mean ± SEM. To compare metabolite profile averages between biological conditions, a mixed model was used with animals as the random effect and groups as the fixed effect, where p < 0.05. For the differential methylation analysis, a logistical regression for replicate statistical testing was used and corrected using the Benjamini-Hochberg method with p* < *0.05 and an FDR of 5%. For gene ontology analyses, gene ontology enrichment was filtered with either a p-value < 0.05 or an adjusted p-value < 0.05.

## Supplementary information

Supplmental figure 1 & 2.

Supplemental tables 1–11.

## Data Availability

Raw sequencing files are available at the European Nucleotide Archive under accession number E-MTAB-8191.
