# Supplementary material for: Metabolism-associated genome-wide epigenetic changes in bovine oocytes during early lactation
Source: Sci Rep. 2020 Feb 11;10:2345. doi: 10.1038/s41598-020-59410-8 (PMC7012839; doi:10.1038/s41598-020-59410-8)
Supplement: Supplementary file 1 — Supplmental figure 1 & 2. [file 41598_2020_59410_MOESM1_ESM.pdf]

Metabolism-associated genome-wide epigenetic changes in bovine oocytes during early lactation

Mikhael Poirier, Dawit Tesfaye, Tsige Hailay, Dessie Salilew Wondim, Samuel Gebremedhn, Franca Rings, Christiane Neuhoff, Karl Schellander, Michael Hoelker.

a

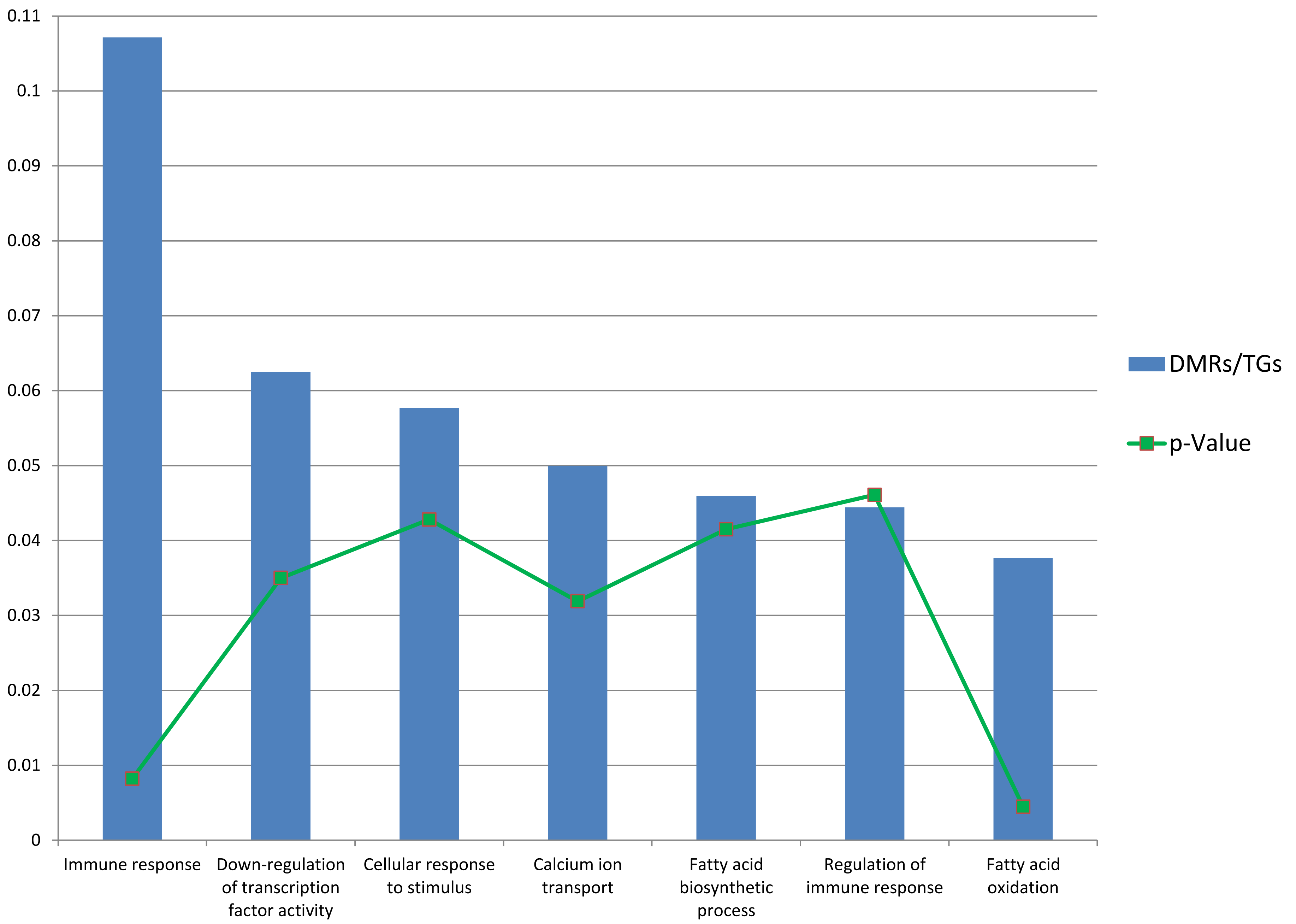

b

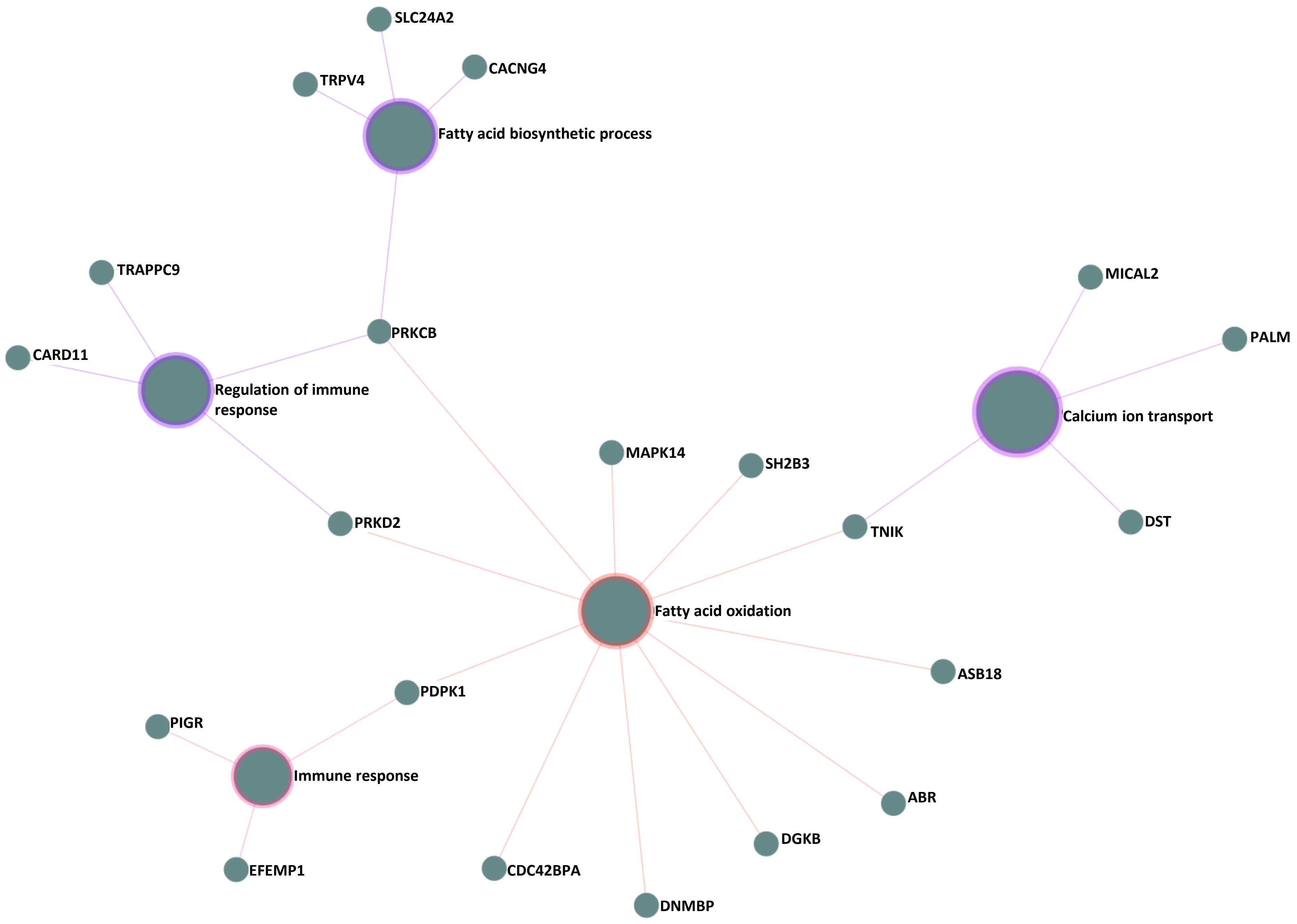

Supplemental figure 1. Functional annotation of mid postpartum (Mpp) specific DMRs. (a) Top biological processes enriched with the DMRs found in the Mpp group. (b) Biological processes network associated with DMRs from the Mpp group and their interaction. DMRs/TGs: Ratio of differentially methylated regions over the number of genes in the pathway. Pathways shown have p-values < 0.05.

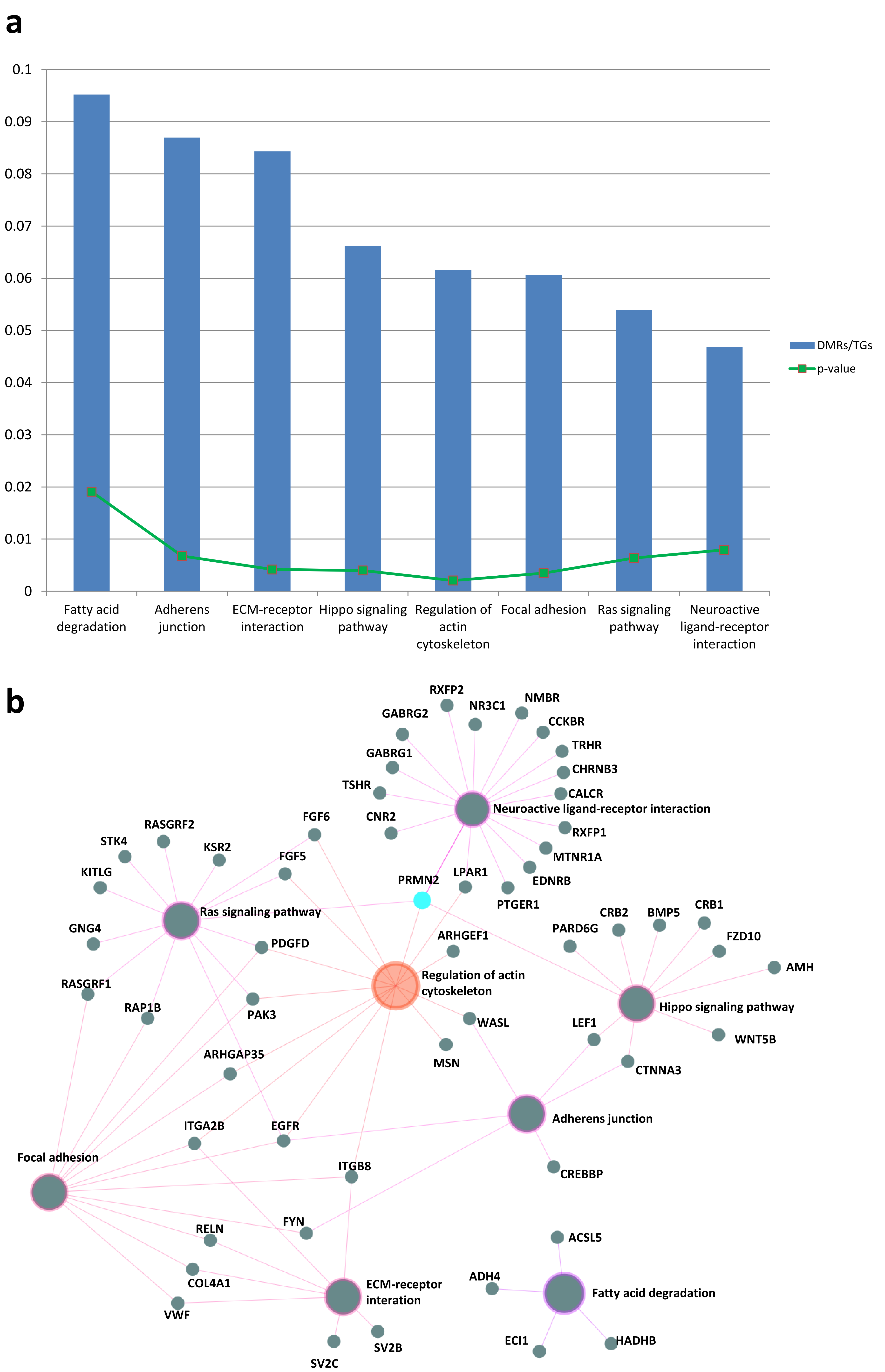

Supplemental figure 2. Functional annotation of cyclic heifer (CH) specific DMRs. (a) Top enriched KEGG pathways associated with the DMRs found in the CH group. (b) KEGG pathway network association with DMRs from the CH group and their interaction. DMRs/TGs: Ratio of differentially methylated regions over the number of genes in the pathway. Pathways shown have p-values < 0.05.
